# Assessment of the Quality of Life during COVID-19 Pandemic: A Cross-Sectional Survey from the Kingdom of Saudi Arabia

**DOI:** 10.3390/ijerph18030847

**Published:** 2021-01-20

**Authors:** Fahad D. Algahtani, Sehar-un-Nisa Hassan, Bandar Alsaif, Rafat Zrieq

**Affiliations:** 1 Department of Public Health, College of Public Health and Health Informatics, University of Ha’il, Ha’il 81451, Saudi Arabia; f.algahtani@uoh.edu.sa (F.D.A.); B.alsaif@uoh.edu.sa (B.A.); r.zrieq@uoh.edu.sa (R.Z.); 2 Molecular Diagnostic & Personalized Therapeutic Unit, University of Ha’il, Ha’il 81451, Saudi Arabia

**Keywords:** quality of life, COVID-19 pandemic, lockdown period, social, environmental, risk factors

## Abstract

The COVID-19 outbreak emerged as an ongoing crisis at the beginning of the year 2020. Its horrific manifestation at the community level significantly affects various dimensions of the quality of life (QoL) of all individuals. The study aimed to examine some of the predictors of the QoL during the first wave of the COVID-19 pandemic in Saudi Arabia. A cross-sectional online survey questionnaire was used to gather data on the participants’ sociodemographic backgrounds, physical health status, psychological reactions, and QoL. We adapted 12 items from the World Health Organization Quality of Life Instruments (WHOQOL-BREF) to assess the QoL. The Depression, Anxiety and Stress Scale–21 (DASS-21) was used to assess depression, anxiety, and stress. The median and inter-quartile range were used to describe the QoL scores. A multinomial regression analysis was computed between QoL score quartiles and associated factors, and the statistical significance was set at *p* < 0.05. The results of the multinomial regression analysis demonstrated that males (OR = 1.96; 95% CI = 1.31–2.94); participants aged 26 to 35 years (OR = 5.1; 95% CI = 1.33–19.37); non-Saudi participants (OR = 1.69; 95% CI = 1.06–2.57); individuals with chronic diseases (OR = 2.15; 95% CI = 1.33–3.48); those who lost their job (OR = 2.18; 95% CI = 1.04–4.57); and those with depression (OR = 5.70; 95% CI = 3.59–9.05), anxiety (OR = 5.47; 95% CI = 3.38–8.84), and stress (OR = 6.55; 95% CI = 4.01–10.70) were more likely to be in the first quartile of the QoL scores. While the full model predicting the total QoL score was statistically significant (R^2^ = 0.962, F (750, 753) = 16,705.4, *p* < 0.001), the three QoL dimensions explained 0.643, 0.283, and 0.036 of the variability in environmental, social, and religious/spiritual dimensions, respectively. The COVID-19 pandemic has significantly influenced various aspects of individuals’ QoL, as well as their physical and psychological health. Community-based interventions are needed to mitigate the pandemic’s negative effects and enhance the health and QoL of the general population.

## 1. Introduction

Coronavirus Disease 2019 (COVID-19), which was first detected in December 2019 in Wuhan, China, has created a public health emergency worldwide [1]. The World Health Organization (WHO) declared COVID-19 a pandemic on 11 March 2020. The pandemic as affected more than 200 countries globally, and has severely affected global health [1]. In the Kingdom of Saudi Arabia (KSA), the first case was detected on 2 March 2020, and the number of cases progressively increased in April 2020. The Saudi government adopted a proactive approach to timely control the spread of the disease. The Ministry of Health launched several educational campaigns on hand hygiene and the adoption of social distancing measures [2]. The delivery of facemasks, gloves, and sanitizers in most public places was ensured since the beginning of the pandemic. The government implemented long curfew hours and lockdowns between March and June 2020 in most KSA regions, and canceled visits to holy places and mosques in the KSA and tourist areas [3]. Educational institutions and most other organizations are still implementing remote work while easing back to normal life.

In the past decade, the quality of life (QoL) has been explored primarily in studies focusing on noncommunicable and chronic diseases. It has been defined as “a patient’s general subjective perception of the effect of illness or medical condition on various domains including physical, psychological, social, and occupational functioning” [4]. QoL assessment across various domains helps identify the range of problems that can influence people’s everyday lives. Literature suggests that QoL is a significant predictor of persistence in overall health and well-being [5]. Outbreaks of infectious diseases, such as COVID-19, negatively affect the physical, social, and psychological functioning of individuals and societies, and have significant economic consequences [6,7]. A previous study from Hong Kong that assessed the health-related quality of life (HRQoL) among survivors of severe acute respiratory syndrome (SARS) six months after the start of the pandemic reported significant impairment in HRQoL for general health domains, physical conditions, and social functioning. A recent study from Morocco reported negative effects of the COVID-19 pandemic on HRQoL [8]. Implementing preventive measures affects people’s daily life activities and certainly influences individuals’ general functioning and well-being [6,9]. Previous research studied the negative psychological effects of quarantine after the SARS outbreak, and recent studies have introduced the term “coronaphobia” to describe stress and anxiety among general populations [10,11,12]. Mental health experts expressed concerns about the repercussions of the COVID-19 pandemic on communities’ psychological functioning and well-being [12,13]. Though recent discussions in literature have cautioned about the broader psychological implications of massive quarantine to control COVID-19 spread on individuals’ QoL [13], the empirical research on the effects of the COVID-19 pandemic on various dimensions of QoL in different countries is scarce.

The World Health Organization (WHO) conceptualized QoL as “an individual’s perception of their position in the life in the context of the culture in which they live and in relation to their goals, expectations, standards and concerns” [14]. The COVID-19 outbreak in the KSA and in many other regions of the world was declared a public health emergency associated with high mortality and morbidity rates, inducing traumatic experience at the collective level [15]. Strict adherence to preventive measures, including wearing facemasks, frequent handwashing, disinfecting surfaces, and above all, maintaining social distancing and quarantine of infected individuals, has significantly influenced people’s daily life activities [15]. During the lockdown period, people in their homes generally depend on cyber resources for academic and professional activities. According to some estimates of Internet usage in the USA, the use of gadgets, such as smartphones, tablets, and laptops, increased by almost 20% during the lockdown period [16]. A study from the KSA reported that students taking virtual classes during the KSA’s early pandemic period experienced moderate to high stress levels [17]. Most social events and professional activities that involved either travel or gathering were canceled. A study conducted during the early pandemic period in China reported moderate to high levels of fear and apprehension associated with the COVID-19 pandemic [18]. Quarantined individuals are at an increased risk of experiencing a wide range of negative emotions, such as fear, anger, guilt, and feelings of loss of control. Due to these reactions, people are likely to feel less safe in their physical environments, and they may be practicing hand/face hygiene, disinfecting surfaces, and engaging in other health behaviors excessively [19]. The case counts for COVID-19 in the KSA reached 275,905 as of 31 July 2020, and 81,680 new cases were reported in the month of July alone. The average daily cases in the month of July 2020 was around 2500. Few studies have demonstrated that patients with chronic health conditions, such as breast cancer, Parkinson’s disease, and those awaiting surgery, experienced deteriorated QoL, particularly in physical and psychosocial dimensions [20,21,22,23].

Therefore, it is important to understand the repercussions of physical health status, social restrictions, and psychological states of people during the COVID-19 outbreak. We believe that pandemic consequences extend to various dimensions of QoL, including environmental, social, and spiritual/religious aspects. Our study aimed to assess these dimensions of QoL in the wake of the COVID-19 outbreak in the KSA, and to determine the association of sociodemographic factors, general physical health, and psychological health with QoL. Our study focused on identifying factors that could decrease the QoL in the general population during the COVID-19 pandemic in the KSA.

## 2. Materials and Methods

### 2.1. Study Settings, Participants, and Procedure

The online cross-sectional survey was conducted between 4–17 July 2020. The target population for this study was the general population residing in the KSA. Further inclusion criteria were (1) being male or female, (2) being 18 years of age and above, and (3) being able to self-report by completing an anonymous online survey questionnaire. A non-random convenience sampling method was employed to select the participants. The minimum sample size was 737, as calculated by $\frac{z^{2}P\left(1-P\right)}{d^{2}}$, assuming that the proportion of psychological distress was 23.6% with a 95% confidence interval (CI) and 0.035 precision [23].

The online survey was the most feasible way to access the target population in light of the social-distance protocols implemented during the COVID-19 pandemic. To recruit the participants from 13 geographical regions of the KSA, we circulated the online survey link through a professional and social network of research team members (focal persons) in these regions. To maximize the reach, the study-invitation link was shared through various online communication channels, including email, organizational portals, and social media platforms (WhatsApp, Twitter, and Facebook).

### 2.2. Survey Questionnaire

The online questionnaire was part of a wider research project to assess individuals’ psychological reactions and quality of life during the COVID-19 pandemic and its associated factors. It measured several areas: (a) sociodemographic data, (b) employment status, (c) health status (e.g., chronic medical conditions), (d) psychological reactions (i.e., depression, anxiety, and stress levels), and (e) the effects of COVID-19 on QoL during the period of lockdown in the KSA. The participants could choose to complete either the English or Arabic version of the survey questionnaire.

#### 2.2.1. Sociodemographic and Health Status Data

The survey assessed sociodemographic characteristics by asking the participants to report their gender, age, marital status, education, job status, nationality, and region of residence. The participants were also asked whether they had any of the listed chronic medical conditions, e.g., diabetes, heart diseases, hyper-cholesterol, arthritis, etc.

#### 2.2.2. Psychological Reactions

The shorter version of the Depression Anxiety Stress Scales (DASS-21) was used to assess the symptoms and levels of stress, anxiety, and depression [24]. Each subscale included seven items rated on a 4-point Likert scale ranging from 0 (did not apply to me) to 3 (applied to me very much) that assessed the levels of depression, anxiety, and stress. The cutoff scores for depression were 0–9 for normal, 10–12 for mild, 13–20 for moderate, 21–27 for severe, and 28–42 for extremely severe levels. For anxiety, the scores ranged from 0–6 for normal, 7–9 for mild, 10–14 for moderate, 15–19 for severe, and 20–42 for extremely severe levels. For stress, the scores ranged from 0–10 for normal, 11–18 for mild, 19–26 for moderate, 27–34 for severe, and 35–42 for extremely severe levels. For the sake of this analysis, the DASS-21 outcome scores were used as dichotomous variables by separating those who had scored in the normal range from those who scored in the mild, moderate, severe, or extremely severe range.

In our study, the DASS-21 scale demonstrated adequate internal consistency, with a Cronbach’s Alpha (α) value of 0.94 and internal reliability coefficients for the stress, anxiety, and depression of 0.89, 0.81, and 0.88, respectively.

#### 2.2.3. Quality of Life (QoL)

To assess the effect of the COVID-19 pandemic on QoL, we adapted 12 items from the WHOQOL-BREF scale [25,26]. Concerned authorities from WHO granted permission to adapt the scale in accordance with the study goals. We selected 12 items with the highest factor loadings on the WHOQOL-BREF scale and modified them to reflect the study’s two objectives: to improve the comprehensibility of items, and to contextualize the QoL to reflect the influence of the COVID-19 pandemic and lockdown measures. The measures used in this online survey were adapted to meet the project objectives. This process included extensive discussion and consultations with public-health experts to discuss the WHOQOL-BREF scale, which can tap into all study variables. Several factors were considered, such as the language and format of the questionnaire, culture, and above all, the context of COVID-19 pandemic. We attempted to address these factors while finalizing the arrangement of items on the study questionnaire, to obtain accurate responses from the prospective participants. Moreover, face validity, as well as focus-group discussions, were conducted to ensure that the meaning of scale items was not changed. The adapted version of the WHOQOL-BREF scale consisted of 12 items, with five point ratings on each item from 1–5; therefore, the lowest possible score was 12, and the highest possible score was 60 for the total scale. Table 1 shows the list of original items and items after adaptation. Low scores represent a lower QoL due to the negative effects of the COVID-19 pandemic. In our research, the QoL measure demonstrated good internal consistency (Cronbach’s alpha = 0.81).

### 2.3. Data Analysis

The QoL scale was not normally distributed; therefore, the median and inter-quartile range were used. A multinomial regression analysis was computed between QoL score quartiles and associated factors in the KSA during the COVID-19 pandemic. Hierarchical multiple regression was used to predict QoL scores for different dimensions during the COVID-19 pandemic. Statistical analyses were performed using SPSS Statistics 22.0 (IBM SPSS Statistics, New York, NY, USA). The level of statistical significance was set at *p* < 0.05.

### 2.4. Ethics

The study followed the ethical principles for research involving human subjects outlined in the Declaration of Helsinki. The Ethics Committee of the University of Ha’il approved the study (ethical approval code: 55456/5/41). All participants voluntarily completed the anonymous survey and gave their informed consent before completing the survey questionnaire. The procedures were clearly explained, and participants could interrupt or quit the survey at any point without explaining their reasons for doing so. Upon completing the online survey form, the participants received an information page listing online counseling and support services.

## 3. Results

The study sample comprised 754 participants. Males constituted 54%, and most (74%) participants were citizens, while the remaining participants belonged to other fifteen nationalities. Most (67%) were married, and the lowest education level was middle school (1.3%), whereas the highest education level was post-graduate (33.6%). In this study, 26% of the participants reported suffering from chronic medical problems. Details about the types of diseases are described in our previous publication [27]. About 55% of the participants suffering from a chronic illness reported avoiding medical visits during the COVID-19 pandemic due to fear of being infected with COVID-19 [27].

### 3.1. QoL

The descriptive analysis of the QoL data during the COVID-19 pandemic in the KSA yielded a minimum score of 12 and a maximum score of 60. The QoL’s median score was 39 and an inter-quartile range (IQR) was 34–43.

### 3.2. Predictors of QoL Score in the First Quartile

Table 2 presents the findings of the multinomial regression analysis between the QoL score quartiles and associated factors during the COVID-19 pandemic. The model was significant at *p* < 0.001, and independent variables explained 30% of the variability in the dependent variable. The findings demonstrated that male participants were almost two times (OR = 1.96; 95% CI: 1.31–2.94) more likely to be in the first quartile (lower scores) compared to females. Participants in the 26–35 age group were five times (OR = 5.1; 95% CI: 1.33–19.37) more likely to be in the first quartile, and participants in the 36–45 age group were almost four times (OR = 3.73; 95% CI: 0.98–14.13) more likely to be in the first quartile compared to the older age group (55–65 years).

The non-Saudi residents were almost two times (OR = 1.69; 95% CI: 1.06–2.57) more at risk to be in the first quartile. Participants who had chronic diseases were two times (OR = 2.15; 95% CI: 1.33–3.48) more likely to be in the first quartile. The participants who lost their job during the pandemic were over two times (OR = 2.18; 95% CI: 1.04–4.57) more likely to have lower QoL scores compared to other groups.

The psychological experiences of the survey participants were found to be significantly associated with lower QoL scores. The findings showed that participants who reported symptoms of depression were about six times more likely to be in the first (OR = 5.70; 95% CI: 3.59–9.05) and the second (OR = 6.01; 95% CI: 3.77–9.59) quartile, and about three times (OR = 2.93; 95% CI: 1.80–4.77) more likely to be in the third quartile. The participants with anxiety were over five times (OR = 5.47; 95% CI: 3.38–8.84), about three times (OR = 3.33; 95% CI: 2.04–4.43), and almost three times (OR = 2.73; 95% CI: 1.64–4.56) more likely to be in the first, second, and third quartile of the QoL scores, respectively. Besides, participants who had stress were over six times (OR = 6.55; 95% CI: 4.01–10.70), almost five times (OR = 4.85; 95% CI: 2.96–7.96), and over two times (OR = 2.58; 95% CI: 1.53–4.37) more likely to be in the first, second, and third quartile, respectively, compared to participants with normal psychological parameters. We used the fourth quartile (the highest score of QoL) as a reference category.

### 3.3. Environment, Social Relations, and Spiritual/Religious Dimensions as Determinants of QoL

Table 3 presents the hierarchical multiple-regression findings predicting the QoL score from different QoL dimensions during the COVID-19 pandemic. The full model that included all QoL dimensions to predict the total QoL score (Model 3) was statistically significant (R^2^ = 0.962, F (750, 753) = 16,705.4, *p* < 0.001). Environmental, social-relation, and religious dimensions explained 0.643, 0.283, and 0.036 of the variability, respectively. Thus, these three dimensions explained 96.2% of the variance in QoL scores during the COVID-19 pandemic.

## 4. Discussion

This study was conducted during the early period of the pandemic in the KSA to investigate the specific effects of the COVID-19 pandemic and lockdown on the various dimensions of QoL. The authorities in the KSA implemented social distancing and lockdown measures between February and June 2020 to contain the spread of new coronavirus infections. However, prolonged lockdown and uncertain experiences have likely had psychological repercussions, as COVID-19 significantly changed many individuals’ daily lives [28,29]. Therefore, it is important to identify individuals at an increased risk of experiencing negative influences of this pandemic on the QoL.

The findings demonstrated that some population segments were more vulnerable to poor QoL during the pandemic due to their demographic backgrounds, job losses, chronic medical conditions, and psychological factors. For instance, male and middle-aged participants were more at risk of lower QoL scores. This increased risk is understandable in the context of the COVID-19 pandemic and lockdown. The QoL scale used in this study covered three major dimensions of QoL: environmental, social, and religious. Environmental items, which assessed participants’ feelings of being safe, access to information, and the pandemic’s effects on income explained almost two-thirds of the variability in the overall QoL score. Simultaneously, the social-relation items, which assessed the pandemic’s effect on maintaining relationships with friends and family, explained more than one-quarter of the variability in the overall QoL score.

On the other hand, the religious-dimension items, assessing the pandemic’s effect on performing religious activities, explained less variability in the QoL score. Concerning the effects of the COVID-19 pandemic on various QoL dimensions, the observations are consistent with the findings of the e-survey, which examined the QoL and the quality of society during the COVID-19 pandemic [30]. This report verified increased concerns in the general population about financial conditions, low levels of trust in institutions, and the pandemic’s repercussions on individuals’ emotional wellness. Given Saudi society’s societal structure and cultural values, male members were more likely to experience lower QoL levels across these dimensions. Males are more likely to work outside their home environments; therefore, they are more likely to feel less safe due to increased exposure. The findings of an online survey of the general population in Italy [9] documented increased levels of anxiety and stress among young people who had to work outside their residence. People employed in workplaces requiring increased contact with others, such as healthcare centers and travel stations, were likely to have higher stress levels due to fear of catching COVID-19.

Male individuals who were the main earners of income for families were likely to be at risk of stress and anxiety due to factors such as poor health or job loss during the pandemic. Furthermore, social distancing restrictions have limited male participants’ social activities, thus influencing their relationships with friends. Moreover, during the lockdown period, the closure of mosques resulted in restricted religious activities. A systematic review study conducted before the COVID-19 pandemic demonstrated the negative effects of social isolation on individuals’ physical health and psychological wellness [31]. During the COVID-19 pandemic, this risk multiplied due to more stringent physical restrictions and isolation as part of primary preventive measures [32]. A web-based survey of people who were quarantined during the 2004 SARS outbreaks in Canada also reported distress and a sense of isolation attributed to the reduced physical contact with friends and family members. The adherence to preventive measures, such as wearing a facemask and social distancing, prohibited them from going to shops to buy food, groceries, and medications, which enhanced their feelings of loneliness [10]. Our study’s findings imply the need to retain social connections and networks during the COVID-19 pandemic, which is possible using the technology that allows individuals to stay in touch with others while complying with the precautionary measures of social distancing. Previous literature has demonstrated the effectiveness of these tools in enhancing children’s resilience at the community level [33].

In our study, non-Saudi nationals were more likely to have lower levels of QoL. The increased susceptibility of expatriates is understandable, considering their social and economic circumstances. Those who rely on daily income cannot earn money during the lockdown and can lose their jobs due to the economic crisis linked to the pandemic. In addition, a larger proportion of expatriates are living away from their immediate or extended families. The economic difficulties and diminished opportunities to visit their families during lockdown are likely to have a negative effect on their relationships with family members. Previous research has also demonstrated that socio-economic marginalization likely increases individuals’ vulnerability to poor physical, psychological, and emotional health and overall QoL [34]. Our study demonstrated that participants who lost their jobs during the pandemic endured the negative effects of COVID-19. Another recent study also evidenced that losing a job during the pandemic led to a higher level of anxiety and depression among non-Saudi residents [35]. Loss of a job during a pandemic can significantly increase the vulnerability to poor QoL. The pandemic can have far-reaching implications on community mental health unless strong supportive measures are taken at the government level to provide psychological and social support to the sufferers. These observations are in line with the existing literature, which has demonstrated that people in certain social groups, such as the age category of 35–49, or those employed for daily wages or self-employed, were financially more vulnerable and likely to experience job insecurity that can take a toll on their QoL [30].

Previous literature has demonstrated that people who have limited or no teleworking experience felt anxiety and stress due to the sudden shift and new demands of this work environment [36]. These findings imply that organizational support should focus on providing social support during crisis time and decreasing the feelings of job insecurity and incompetence among employees. Additionally, to enhance the environmental QoL among employed individuals, organizations can enhance feelings of competence by appreciating their current contributions and willingness to adjust to and accept new work demands, despite the crisis and generalized distress at societal levels during the COVID-19 pandemic. 

Our study findings demonstrated that respondents with chronic medical conditions (hypertension, diabetes, heart disease, cancer, obesity, psychological problems, arthritis, etc.) reported significantly lower QoL scores. The finding can be explained by the fact that people with comorbid medical conditions often require urgent medical attention due to worsening symptoms, or require treatment follow-up. These opportunities were restricted during the lockdown period. Our study results are consistent with the findings of a study from Morocco, which reported largely negative effects of the COVID-19 pandemic on QoL and the well-being of individuals with chronic medical conditions [6]. Another study also supported the vulnerability of individuals with chronic medical problems to experience poor quality of life during the first wave of COVID-19, which explains the finding that “chronic illness or a self-evaluation of poor health is associated with increased psychological distress” [10]. The tendency to avoid medical visits during the pandemic was documented by another study in which more than half (55%) of the survey participants avoided medical visits during the first wave of the COVID-19 pandemic due to fear of COVID-19 infection [27].

In the current era, wide access to technology has increased people’s access to healthcare services and social support to buffer the pandemic’s negative effects on mental health [37]. In Saudi Arabia, the Ministry of Health launched an online portal with several health applications to ensure access to basic healthcare services and fulfill the general population’s pharmacy needs. Nevertheless, the fear and panic associated with the pandemic and lockdown have taken their toll, and people with chronic medical conditions are more likely to experience psychological burden and poor QoL. The findings emphasize the importance of obtaining countrywide data for people with chronic medical conditions to estimate their healthcare and financial needs during the pandemic. This should be an ongoing process that mobilizes the existing community resources to cater to these populations’ specific needs.

Our study demonstrated that participants who had experienced anxiety, depression, and stress were at an increased risk of having lower QoL scores. The psychological dimension is an important component of overall QoL, and the current study’s findings validated its relationship with other QoL dimensions. The observations were somewhat consistent with a previous study from KSA, which reported higher distress levels among people who rated their health as poor [11].

Among the study limitations, we acknowledge that a one-time cross-sectional study could not capture the ongoing effects of the COVID-19 pandemic on various dimensions of QoL; thus, future research could collect the data using a longitudinal design. We did not use a preference-based measure for QoL (e.g., SF-6D or CORE-6D) as these tools are primarily used to assess QoL in clinical populations to determine the impact of specific physical and mental health disorders on the QoL of patients. In our study, the aim was to assess the overall impact of the pandemic on the QoL of the general population, so a short online survey questionnaire was a more appropriate approach to collect data from large segments of general population. Despite an online survey being the best data-collection method during the COVID-19 pandemic due to lockdown measures, the findings need to be interpreted while considering some limitations of online surveys. For instance, the electronic survey link was distributed through social media platforms, email, organizational mailing lists, and the like; therefore, the characteristics of the survey participants are unknown. Voluntary participation in the survey could have resulted in biases, as the respondents might have had easier access to the Internet and felt more comfortable with online tools [38]. A unique factor during the lockdown period of COVID-19 that may have affected the response rate is saturation with online activities, since people increased their screen time to engage in online work, academic work, and even leisure activities. Despite some of the limitations of online studies, our study’s findings provide useful insight into the effects of the COVID-19 pandemic on the QoL and its associated factors.

## 5. Conclusions

Overall, male gender; participants 26 to 35 years of age; non-Saudis; those who lost their jobs; those with chronic diseases; and those who had higher levels of depression, anxiety, and stress were at a higher risk to have lower levels of QoL during the COVID-19 pandemic and lockdown period in the KSA. The environmental QoL explained most of the variance among the three QoL dimensions, followed by the social and religious/spiritual dimensions. The findings thus demonstrated the epidemiological profile of the population more likely to have a lower QoL during the pandemic. Furthermore, the focus should be on improving the environmental and social-relationship domains of QoL, which were most significantly influenced during the pandemic, as the environmental domain explained 64% of the variability and the social-relationship domain explained 28% of the variability. Overall, the findings validated the negative effect of the COVID-19 pandemic on the various aspects of individuals’ QoL and physical and psychological health.

The results strongly suggest the need for community health and wellness programs to be implemented in the coming months to deal with the current and imminent effects of this local and global crisis. These recommendations are also in line with the Saudi Vision 2030, which focuses on fulfilling individuals’ needs to live healthy lives and providing preventative measures to combat the psychological effects of the crisis. The findings provide baseline evidence and highlight the need to replicate the effects of the COVID-19 pandemic in larger samples of individuals with various chronic diseases, and to explore factors related to exacerbation or remission of the disease, possible emergencies, and the need for medical care. These investigations will help tailor community health programs and enhance the general health and well-being of communities in the KSA and other countries affected by the COVID-19 pandemic.

## Figures and Tables

**Table 1:** The list of items adapted to assess QoL in the context of the COVID-19 pandemic.

	Items before Adaptation on WHOQOL-BREF	Items after Adaptation
1	How would you rate your quality of life?	How would you rate the impacts of COVID-19 pandemic on your quality of life?
2	How satisfied are you with your health?	How would you rate impacts of COVID-19 pandemic on your general health?
3	How safe do you feel in your daily life?	How would you rate the impacts of COVID-19 pandemic on your feelings of being safe in your daily life?
4	How healthy is your physical environment?	How would you rate the impacts of COVID-19 pandemic on your physical environment?
5	How available to you is the information that you need in your day-to-day life?	Keeping in view the impacts of COVID-19 pandemic, how available to you was the information that you needed in your daily life?
6	Have you enough money to meet your needs?	How would you rate the impacts of COVID-19 pandemic on your income?
7	How satisfied are you with your access to health services?	How would you rate the impacts of COVID-19 pandemic on your access to health services?
8	How satisfied are you with your personal relationships?	How would you rate the impacts of COVID-19 pandemic in maintaining relationship with your friends?
9	How satisfied are you with your personal relationships?	How would you rate the impacts of COVID-19 pandemic in maintaining relationship with your family?
10	How satisfied are you with the support you get from your friends?	Keeping in view the impacts of COVID-19 pandemic, ‘how satisfied were you with the support you get from your friends?’
11	To what extent does faith give you comfort in daily life?	To what extent does faith give you comfort to deal with hard time of COVID-19 pandemic?
12	To what extent does any connection to a spiritual being help you to get through hard times?	How would you rate the impacts of COVID-19 pandemic on your spiritual connections/practice?

**Table 2:** The multinomial regression between the QoL score quartiles and associated factors in the KSA during the COVID-19 pandemic.

		QoL Quartiles			
		1st	2nd	3rd	4th
		OR (95% CI)	OR (95% CI)	OR (95% CI)	OR (95% CI)
Gender					
Male	408 (54.1)	1.96 (1.31–2.94) **	1.30 (0.87–1.94) ns	0.34 (0.80–1.86) ns	Reference
Female	346 (45.9)	Reference	Reference	Reference	Reference
Age group					
18–25 years	146 (19.4)	3.43 (0.87–13.60) ns	1.63 (0.58–4.58) ns	1.82 (0.59–5.55) ns	Reference
26–35 years	222 (29.4)	5.10 (1.33–19.37) *	1.38 (0.50–3.78) ns	1.67 (0.56–4.95) ns	Reference
36–45 years	257 (34.1)	3.73 (0.98–14.13) *	1.21 (0.45–3.26) ns	1.24 (0.42–3.65) ns	Reference
46–55 years	102 (13.5)	2.62 (0.64–10.68) ns	1.25 (0.43–3.63) ns	0.54 (1.43–4.51) ns	Reference
55–65 years	27 (3.6)	Reference	Reference	Reference	Reference
Nationality					
Non-Saudi	195 (25.8)	1.69 (1.06–2.57) *	1.28 (0.79–2.06) ns	1.29 (0.79–2.13) ns	Reference
Saudi	559 (74.1)	Reference	Reference	Reference	Reference
Education					
Middle School	10 (1.3)	1.10 (1.77–6.75) ns	1.84 (0.32–10.44) ns	0.68 (0.05–6.84) ns	Reference
High School	89 (11)	0.54 (0.28–1.02) *	0.48 (0.23–0.96) *	0.73 (0.37–1.46) ns	Reference
University	401 (53.2)	0.77 (0.50–1.20) ns	1.11 (0.71–0.73) ns	1.22 (0.75–1.95) ns	Reference
Post-graduate	253 (33.6)	Reference	Reference	Reference	Reference
Chronic disease					Reference
Yes	195 (25.9)	2.15 (1.33–3.48) **	2.01 (1.23–3.27) **	1.60 (0.95–2.69) ns	Reference
No	559 (74.1)	Reference	Reference	Reference	Reference
Loss of job					
Yes	51 (6.8)	2.18 (1.04–4.57) *	0.67 (0.26–1.71) ns	0.71 (0.27–1.87) ns	Reference
No	703 (93.2)	Reference	Reference	Reference	Reference
Depression					
Yes	328 (43.5)	5.70 (3.59–9.05) ***	6.01 (3.77–9.59) ***	2.93 (1.80–4.77) ***	Reference
No	426 (56.5)	Reference	Reference	Reference	Reference
Anxiety					
Yes	263 (34.9)	5.47 (3.38–8.84) ***	3.33 (2.04–5.43) ***	2.73 (1.64–4.56) ***	Reference
No	491 (65.1)	Reference	Reference	Reference	Reference
Stress					
Yes	275 (36.5)	6.55 (4.01–10.70) ***	4.85 (2.96–7.96) ***	2.58 (1.53–4.37) ***	Reference
No	479 (63.5)	Reference	Reference	Reference	Reference

OR: Odd ratio. Symbols illustrates the level of statistical significance: ns > 0.05; * < 0.05; ** < 0.01 *** < 0.001.

**Table 3:** The hierarchical multiple regression predicting the QoL score from different QoL dimensions during the COVID-19 pandemic.

Variables	QoL Score					
	Model 1		Model 2		Model 3	
	B	β	B	β	B	β
**Constant**	13.91 ***		4.53 ***		1.51 ***	
Environmental dimension of QoL	2.58 ***	0.68	1.35 ***	0.39	1.11 ***	0.03
Social Relations dimension of QoL		0.07	1.37 ***	0.026	1.23 ***	0.02
Religious/Spiritual dimension of QoL					1.2 ***	0.04
**R**	0.802		0.962		0.981	
**R^2^**	0.643		0.962		0.962	
**F**	1352.7 ***		4690.6 ***		16,705.4 ***	
**∆R^2^**	0.643		0.283		0.036	
**∆F**	1352.7 ***		2896.1 ***		707.8 ***	

B = Non-standardized regression coefficient; β = Standardized coefficient; R^2^ = coefficient of determination; ∆R^2^ = R^2^ change; ∆F = F change; *** < 0.001.

## Data Availability

The data presented in this study are available on request from the corresponding author. The data are not publicly available.

## Declaration

Adaptations made into Arabic language from Arabic-WHOQOL-BREF, Geneva, World Health Organization (WHO), (3 May 2020), (https://www.who.int/toolkits/whoqol/whoqol-bref/docs/default-source/publishing-policies/whoqol-bref/arabic-whoqol-bref, accessed 2 June 2020). WHO is not responsible for the content or accuracy of this translation/adaptation. In the event of any inconsistencies between the English and the Arabic translation, the original English version shall be the binding and authentic version.
